# Microbiological Quality Assessment of Some Commercially Available Breads

**DOI:** 10.3390/foods13203271

**Published:** 2024-10-15

**Authors:** Éva György, Éva Laslo

**Affiliations:** Department of Food Science, Faculty of Economics, Socio-Human Sciences and Engineering, Sapientia Hungarian University of Transylvania, 530104 Miercurea Ciuc, Romania; lasloeva@uni.sapientia.ro

**Keywords:** microbiological quality, bread, public safety, allochthonous microorganisms

## Abstract

Bread is a staple, energy-rich food for people of all ages, so quality is important to consumers. In our region, most of the commercially available bread, whether packaged or unpackaged, is produced by local bakeries, so monitoring microbial levels and the types of microbes present on bread can help to draw attention to protect the final product. It can also help to ensure the food safety, quality, and shelf life of bread. The freshly baked product is microbiologically sterile. Post-process contamination affects the microbial load of bread. In this study, the microbial load of 30 different commercial bread crumbs and crusts was determined. The different types of bread with different compositions were analyzed for total viable bacteria, *Escherichia coli*, *Staphylococcus aureus*, aerobic and anaerobic spore-forming bacteria, and culturable microscopic fungi. The K-means clustering algorithm was used to cluster the different types of bread based on the number of aerobic mesophilic bacteria. Significant differences (*p* < 0.05) were found in the total viable bacterial count for bread crusts and crumbs. The bacterial count of bread varied between 10.00 ± 0.00–395.00 ± 52.4 CFU/g for bread crusts and 10.00 ± 0.0–310.67 ± 94 CFU/g for bread crumbs. The results of 16S rDNA sequence analysis showed that the most frequently occurring bacterial species belonged to the genus *Bacillus*, but species of the genus *Staphylococcus* were also present. *Chryseobacterium* spp. predominated on multigrain bread, *Marinilactobacillus* spp. on rustic potato bread, and *Staphylococcus warneri* on sliced brown potato bread. The results contribute to a better understanding of the microbial dynamics in locally produced breads from the Eastern Carpathians of Transylvania, with the aim of improving food safety, quality control, and consumer protection.

## 1. Introduction

One of the staples of the diet of ancient civilizations was flat bread/pie, which evolved over time into bread. It is thought to have originated in Mesopotamia, during the Sumerian culture, from where it spread to Europe. Wheat has played a central role in the history of the European diet. In modern Europe, loafy breads with a loose grain became widespread, the quality of which was largely determined by the quality of the cereals used, their physical and chemical properties, and the quality of the leaven.

In the second half of the 20th century, very few households baked bread in large quantities, and bakers and bread factories increasingly took over this task from the housewife [1,2].

Bread is a typical bakery product with many varieties, made from flour of different cereals with additives and yeast [3]. Bread is preserved by baking. In general, the shelf life of bread is 5–7 days at room temperature, 1–2 weeks in the refrigerator, and 3 months in the freezer.

Bread microbial spoilage is caused by microbial contamination after baking. The source of microbial contamination is the air and the equipment used during handling operations such as cooling, slicing, and packaging. The number and type of microorganisms present on bread depends on the production environment and the hygiene of the equipment used. Microorganisms can also originate from ingredients. Personal hygiene is also considered to be of paramount importance [4,5,6,7,8,9,10].

Bread spoilage microorganisms belong to different genera: *Bacillus*, *Clostridium*, *Lactobacillus*, *Leuconostoc*, *Aspergillus*, *Penicillium*, *Cladosporium*, *Claviceps*, *Rhizopus*, *Candida*, *Saccharomyces*, and *Zygosaccharomyces* [4,5,6,7,8,9,10]. Some of them cause ropiness, moldiness, and off-flavors.

Ropiness appears in non-acidified bread because organic acids present in sourdough bread made with kefir grains can inhibit *Bacillus* spp. species [6,11].

Within the endospore-forming bacteria *B. subtilis*, *B. amyloliquefaciens*, *B. pumilus*, *B. megaterium*, *B. licheniformis*, *B. claussi*, *B. firmus*, and *B. cereus* are associated with bread ropiness [11,12,13,14]. Recently, *B. velezensis*, *B. spizizenii*, and *B. inaquosorum* have also been associated with the spoilage of bread [10].

After freezing, during thawing, the bread absorbs moisture due to ice crystals that break up, which is sufficient for the development of yeasts and bacteria that cause spoilage and a sour taste. Due to the growth of mucoid strains of *B. subtilis*, bread becomes soft, sticky, brown, and has an unpleasant fruity odor. The endospores of the above-mentioned bacteria, which originate from raw materials such as flour or bakery equipment, survive the baking process and germinate within 1–2 days. They also produce extracellular amylases and proteases and alter the structure of the bread. High moisture content in the dough, slow cooling, and pH > 5 favor bread spoilage [15,16,17,18]. Industrial wholemeal flours contained high levels of aerobic endospore-forming bacteria, reaching 3.1 log spores/g. The identified bacteria were *B. licheniformis*, *B. sonorensis*, *B. cereus*, *B. pumilus*, and *Paenibacillus polymyxa*. These included amylase producers and contributed to the spoilage of preservative and preservative-free bread [14].

*Serratia marcescens* is responsible for the development of bloody bread as a result of pigment release [5]. Different species of lactic acid bacteria belonging to *Lactobacillus* genus as *L. plantarum*, *L. curvatus*, *L. casei*, *L. farciminis*, *L. alimentarius*, *L. sanfranciscensis*, *L. fermentum*, *L. brevis lindneri*, *L. fructivorans*, *L. buchneri*, *L. acidophilus*, or members of genus *Pediococcus*, *Carnobacterium*, *Enterococcus*, *Oenococcus*, *Streptococcus*, *Tetragenococcus*, *Vagococcus*, *Weisella* contribute to the development of flavor defects [5].

In sourdough bread, lactic acid bacteria such as *L. brevis*, *L. plantarum*, and *L. sanfranciscensis* contribute to the reduction in bacterial and fungal spoilage of the product and also influence the properties of the bread crumb [19]. Olive leaf extract and lactic acid bacteria increased the shelf life and quality of sourdough bread. Positive effects were achieved in improving moisture content, pH, acidity, softness, texture, flavor, odor, staleness, and chewiness [20].

Preservative-free bread becomes moldy after 5–6 days. If yeasts are present, white or pink spots may appear on the surface of the bread. Some molds can produce mycotoxins [4,5,6,7]. Several factors determine the presence of mold. Compared to the higher water content of bread crumbs, the crust contains only 16% moisture, which is a major protection against molds. Sliced and packaged bread is more susceptible to mold growth. The most characteristic microscopic fungi were species of the genera *Penicillium*, *Aspergillus*, *Cladosporium*, *Mucor*, and *Rhizopus* [4]. In wheat bread were detected *Penicillium commune*, *P. solitum*, *P. corylophilum*, and *Aspergillus flavus*, whereas in rye bread *P. roqueforti*, *P. corylophilum*, and *Eurotium* species were detected in higher numbers. Common bread spoilage fungi as *Aspergillus flavus*, *P*. *commune*, *P. roqueforti*, and *Endomyces fibuliger* were inhibited by spices and herbs due to their antimicrobial compounds. Essential oils were effective in inhibiting the molds, while mustard, garlic, clove, and oregano oleoresin were less effective. *P. roqueforti* was the most sensitive, while *A. flavus* showed resistance to the essential oils tested [21,22]. Predominant species of *A. niger* and *P. sumatrense* in pan bread were inhibited by the essential oil of *Tahiti lemon* [23].

The water activity of various types of bread is usually low enough (a_w_ = 0.75–0.9) to prevent bacterial growth. However, certain molds, such as *Rhizopus stolonifer*, often found on bread, can grow if the moisture content increases during storage due to starch crystallization. Baking yeasts inhibit mold growth, but spores can be transferred to bread from the air and equipment [8,15,16,17,18]. The bread-making technology (combined radio frequency, 58 °C hot air treatment) also helps to extend the shelf life of white bread and reduce *P. citrinum* spores by four logs [24]. The precautionary measure against fungal contamination is the packaging system. [25]. A wide range of bread packaging systems are used, from traditional paper to innovative functional packaging solutions. Chitosan and beeswax–chitosan-coated paper bread packaging has been shown to improve sensory and analytical properties under refrigerated conditions [26]. According to Wang et al. (2023) [27], the application of an antifungal bilayer film to bread with cinnamaldehyde-loaded polylactic acid, konjac glucomannan, and wheat gluten reduced the incidence of *Aspergillus* and *Penicillium* species.

According to these findings, breads can pose health risks to consumers and lead to significant economic losses. The spoilage of these products can be a food safety problem. There is a lack of scientific information on the microbiota of breads from the Eastern Carpathians region of Transylvania. The aim of this study was the microbiological analysis and detection of allochthonous microorganisms in various commercially available breads.

## 2. Materials and Methods

The microbiological load of 30 different commercial bread samples was determined using cultivation methods on different selective media. The analyzed bread types belonged to multigrain bread, rustic potato bread, sliced white bread, white bread, rye bread, sliced brown barley bread, sliced brown potato bread, peasant bread, wheat germ bread, wholemeal bread, Graham bread, bran bread, and French bread. The ingredients of the most analyzed breads were listed on the product labels. All breads contained the main ingredients, such as wheat flour, water, yeast, and salt. In addition to the basic ingredients, different ingredients were used for the traditional or special types of bread. Multi-grain breads contained different seeds, such as pumpkin seeds, sunflower seeds, sesame seeds, and linseed. Rustic potato breads contained potatoes in the form of paste or flakes. White bread contained vegetable oil, while rye bread contained rye flour. Farmers’ bread contained sourdough. Wheat germ bread was made with wheat germ. In addition to flour, the following breads contained other types of flour: wholemeal bread contained wholemeal flour, graham bread contained graham flour, and bran bread contained wheat bran. The freshly bought breads were taken to the laboratory and sampled within 1 h under aseptic conditions using sterile utensils. Three different samples were also taken from each bread sample crumbs and crusts.

The total number of aerobic mesophilic bacteria was determined on nutrient agar medium (HiMedia) using the pour plating method. Moreover, 25 g of each bread crumb and crust sample was transferred into a 225–225 mL sterile physiological solution. A volume of 1 mL of each suspension was used for total aerobic mesophilic bacteria count determination. Incubation was carried out at 37 °C for 48 h. The presence of aerobic endospore-forming bacteria was determined on ChromoBio^®^ Cereus Selective Base Agar (Biolab) medium, with the difference that the above-mentioned sample suspensions were heat treated at 80 °C for 10 min. Incubation was carried out at 37 °C for 48 h.

The presence of anaerobic spore-forming *Clostridium perfringens* was detected in Clostridial Differential Broth (Biolab). In 10 mL of Clostridial Differential Broth, it was added paraffin and 1 mL of each bread crumb and bread crust suspension, and it was incubated for 10 min at 80 °C in a water bath. After cooling, it was incubated for 48 h at 37 °C.

The presence of the personal hygiene indicator bacteria *Escherichia coli* and *Staphylococcus aureus* was determined on the selective TBX Chromo Agar (Oxoid) medium and Mannit–Kochsalz Agar (Carl Roth) medium with the spread plate method and incubation at 37 °C for 48 h.

The presence of culturable microscopic fungi was determined on Czapek Dox (Sigma) Agar medium with the spread plate method, with incubation at 25 °C for 4–5 days. The morphological identification of the fungi was carried out using microscopic slides [28,29].

Bacterial colonies with the highest number and characteristic colony morphology were isolated from the different agar media, and pure cultures were made. The isolated bacterial strains were identified by 16S rDNA gene sequence analysis.

Genomic DNA was isolated according to the AccuPrep^®^ Genomic DNA Extraction Kit (Bioneer) protocol. Moreover, 27f 5′ AGAGTTTGATCMTGGCTCAG 3′ and 1492r 5′ TACGGYTACCTTGTTACGACTT 3′ universal oligonucleotides were used for the amplification of the bacterial 16S rDNA gene. The PCR reaction included an initial denaturation at 94 °C for 5 min, which was followed by 30 cycles of denaturation at 94 °C for 30 s, annealing at 55 °C for 30 s, extension at 72 °C for 1 min, and a final extension at 72 °C for 7 min.

The amplified PCR products sequencing of bacterial isolates was performed by the commercial service of Biomi KFT (Hungary). The resulting sequences were edited and aligned with Chromas version 2.6.6, and the Molecular Evolutionary Genetics Analysis 4 system was used for phylogenetic analyses [30,31]. The isolated bacteria were identified through comparison of the sequences using the EzTaxon server based on the 16S rDNA sequence data [28,32].

### Statistical Analysis

Results were analyzed using K-mean cluster analysis, one-way ANOVA followed by post hoc Duncan’s test, and Bonferroni using IBM SPSS Statistics v22. *p* < 0.05 was considered statistically significant.

## 3. Results and Discussion

Based on our results, the microbiological quality of the bread samples tested is diverse. The bread samples come from a variety of bakeries in two different counties in the region. The aerobic mesophilic total viable count on nutrient agar medium was highly variable among the breads tested, both for crumb and crust samples (Table 1). A high total viable count indicates a high level of microorganisms, which can have several consequences. The highest number of mesophilic bacteria was detected in the crust of the rustic potato bread crust: 395.00 ± 52.43 CFU/g. High total plate counts were also found in the crusts of multigrain bread 1 (164.00 ± 100.59 CFU/g), sliced white bread 1 crumb (310.67 ± 94 CFU/g), rye bread 1 (239.00 ± 235.20 CFU/g), and graham bread 2 (206.00 ± 32.4 CFU/g). Low plate counts of aerobic bacteria were found in multigrain bread 2 crumb (10.00 ± 0.00 CFU/g), multigrain bread 6 crumb (10.33 ± 0.58 CFU/g), white bread 3 wp crumb (10.67 ± 1.15 CFU/g), rye bread 2 crumb (10.00 ± 0.00 CFU/g), peasant and bran bread crumb (10.00 ± 0.00 CFU/g), and French bread crumb (10.33 ± 0.58).

Bread crumb samples have lower cell counts than crust samples in several cases, but in samples with high total plate counts, crust and crumb showed similar results, e.g., sliced white bread 1.

Based on the K-means clustering algorithm, the different types of bread based on variables of aerobic and mesophilic bacteria count from different parts of the bread crumb and crust were clustered. The iteration history shows that four clusters were able to converge to zero change after three iterations, which is a good sign that four clusters are a stable, strong cluster solution. Based on the results, there are seven respondents in cluster 1, three in cluster 2, seventeen in cluster 3, and three in cluster 4. The very small clusters are also significant; they are underrepresented. Cluster 3 has a good, reasonable size, and this cluster is the most represented.

In the case of ANOVA in this case (Table 2), the F values are taken into account, and it can be affirmed that the value of F reveals that statistics are not very strong but the p-values are significant. The aerobic mesophilic bacteria count of bread crust has a greater influence in deciding the cluster, where the F value is 70.34 and the aerobic mesophilic bacteria count of bread crumb is the least important variable with a F value of 55.675.

**Figure 1:**
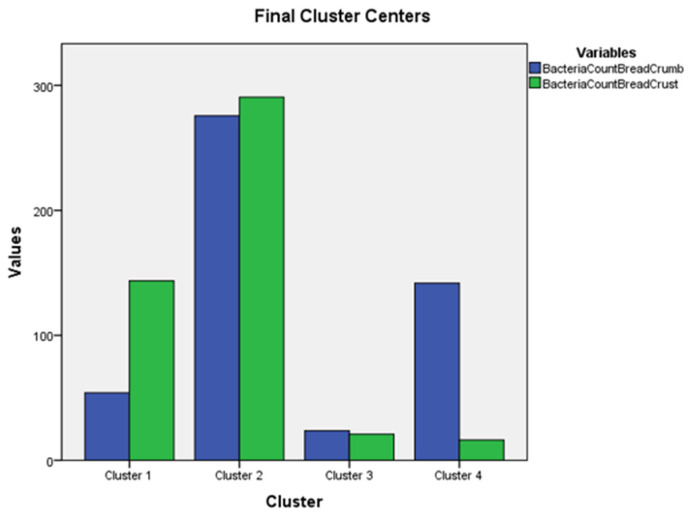
The graph of final cluster centers.

The final cluster centers (Figure 1) showed that in cluster 2, the average bacterial count of the bread crust respondents was 290.56, while the bacterial count of the bread crumb was lower, equal to 275.67.

In cluster 3, the respondents’ average bread crust bacterial count is 20.94, while the bread crumb bacterial count is 23.63. In cluster 4, an average of the respondents’ bacteria count of bread crust is high (141.78), while the bacteria count of bread crust is low (16.22). In cluster 1, the respondent’s bacteria count of bread crust is 54.09, and the bacteria count of bread crust is high (143.62).

To determine if there were significant (*p* < 0.05) differences between the cluster variables (Figure 1), the four cluster variables were compared using a one-way ANOVA and post hoc Bonferroni tests (Table 3).

Cluster analysis was used to categorize samples in groups based on a variety of variables [33]. As a result of the different total viable counts in bread crust and bread crumb, even within one bread, led to the use of cluster analysis to characterize the group of samples analyzed (three different samples/breads were taken from each type of bread in both cases, crust and crumb). K-mean clustering was used to compare whether the variables within a cluster showed significant differences. The multiple comparison showed that the bacterial count of bread crumbs for cluster 1 compared to clusters 2 and 4; cluster 2 compared to clusters 1, 3, and 4; cluster 3 compared to clusters 2 and 4; and cluster 4 compared to clusters 1, 2, and 3 are significantly different (*p* < 0.05). The difference between cluster 1 and cluster 3 is not significant, *p* = 0.295.

The multiple comparison showed that bread crust bacteria count for cluster 1 compared to clusters 2, 3, and 4; cluster 2 compared to clusters 1, 3, and 4; cluster 3 compared to clusters 1 and 2; and cluster 4 compared to clusters 1 and 2 are significant differences (*p* < 0.05). Only cluster 3 compared to cluster 4 on variable bread crust bacterial count was not significant, *p* = 1.

Based on the K-mean clustering approach, we obtain different clusters of the different bread types based on the bacterial count. K-means is the most widely used partitioning clustering technique in food science and technology. Nurlaila et al., 2021 [34] applied K-means clustering to group bacteria into clusters based on functional phenotypic characteristics.

Aerobic endospore-forming bacteria are present in bakery processing environments and may pose a threat to the safety and quality of bread. Among the total aerobic spore-forming bacteria, *B. cereus* was not present in the bread samples tested.

The results obtained on selective agar medium were as follows: *B. subtilis* was present in the following samples: multigrain bread 1 (1 × 10 CFU/g), rustic potato bread 2 (1.3 × 10^2^ CFU/g), sliced white bread 1 (3 × 10 CFU/g), rye bread 1 (4 × 10 CFU/g), and rye bread 2 (2 × 10 CFU/g). *B. subtilis* was present in the following bread crust samples: rustic potato bread 1 (1.3 × 10^2^ CFU/g), rustic potato bread 2 (4 × 10 CFU/g), and sliced white bread 1 (2 × 10 CFU/g).

*Clostridium perfringens*, belonging to the group of anaerobic sulphite-reducing spore-forming bacteria, was present in one sample of rustic potato bread 1, from the surface and crumbs of the bread. *S. aureus* was isolated on selective medium from multigrain bread 1 (3 × 10 CFU/g), rustic potato bread 1 (2 × 10 CFU/g), rustic potato bread 2 (1.1 × 10^2^ CFU/g), rustic potato bread 4 (3.4 × 10^2^ CFU/g), sliced white bread 1 (1.3 × 10^2^ CFU/g), rye bread 1 (2.3 × 10^2^ CFU/g), and sliced brown potato bread (1 × 10 CFU/g). It was confirmed that the absence of gloves and the practice of displaying bread outside the shop were strongly associated with *S. aureus* contamination [13,35].

The breads analyzed were free from the hygienic indicator bacteria *Escherichia coli*. Microscopic fungi were present in three multigrain bread samples (1, 4, and 6), five rustic potato bread samples (1, 2, 3, 5, and 6), three rye bread samples (1, 2, and 4), sliced barley bread, sliced brown potato bread, French bread, and wholemeal bread. The isolated molds, based on microscopic examination, belong to the genera *Aspergillus* and *Penicillium*. This supports the conclusions of Katsi et al., 2021 [36], who revealed that bread spoilage may be caused by these microscopic fungi. Martins et al., 2021 [37] also observed the presence of *Aspergillus* spp. and *Penicillium* spp. in pearl millet sourdough bread stored in different packaging materials at different temperatures, suggesting cross-contamination within the production zone. These microorganisms can alter the sensory properties of bread and pose safety risks through the production of mycotoxins [13].

The results obtained show that the peasant bread was characterized by the best microbiological quality. Due to the high baking temperatures, the bread is considered a sterile product. The microbial contamination of the bread results from post-baking processes such as cooling, slicing, packaging, and transport [13,36,38]. According to the result of 16S rDNA sequence analysis, bacterial isolates from different breads belong to seven genera with 99–100% similarity. The most common bacterial species belong to the genus *Bacillus*. The second most common bacterial species belongs to the genus *Staphylococcus*. The results of the identification of the bacterial strains isolated from the different types of bread are summarized in Table 4.

Under favorable conditions, bacterial growth and proliferation are influenced by bread ingredients such as starch and proteins during production or storage [7,29]. There are differences between bread types with different compositions, plate counts, and isolated species. Multigrain bread 1 and multigrain bread 6 with pumpkin seeds were found to have high viable cell counts compared to other multigrain breads where other ingredients were listed in addition to this cereal. In the case of multigrain bread 3, sunflower seeds and sesame seeds were listed, and the viable cell count of this bread crumb and crust was medium compared to the other multigrain breads. It should be mentioned that multigrain bread 3 contains potato paste or flakes. The crust of multigrain bread 4 with toasted wheat malt flour and bran had a higher viable cell count. Valková et al., 2021 [39], confirmed that breads supplemented with seed micropowder had a lower total viable cell count than the control bread. Bacteria isolated from multigrain bread 1 were *Chryseobacterium* spp., *B. tequilensis*, *B. subtilis*, and *B. subtilis* subsp. *inaquosorum*. *Chryseobacterium* with low incidence have been found in durum wheat sourdoughs and are mentioned as a part of the wheat flour microbiota [13,40]. It has been confirmed that *B. subtilis* endospores can survive baking and germinate. The endospores form inside the bread within 36–48 h, resulting in a peculiar fibrous, soft, brown mass with a fruity odor, resulting in the formation of volatile compounds such as isovaleric aldehyde, diacetyl, acetoin, and acetaldehyde [7].

Of the breads containing potatoes, rustic potato bread 2 and rustic potato bread 5 had high viable cell counts, as did rustic potato bread 1 and sliced brown potato bread crust. Only in these two cases was it likely that the potato contributed to a higher multiplication of the bacterial cell count. Also, in the case of the potato breads, the increased starch content favored the *Bacillus* species. In the different rustic potato breads, the bacteria identified were *Bacillus* spp. (Table 4). From rustic potato bread 1 among the *Bacillus* spp. (*B. siamensis* and *B. amyloliquefaciens*), it was also detected *Staphylococcus warneri*.

*B. subtilis* and *B. pumilus* with low temperature tolerance; *S. warneri* and *S. aureus* were also present in puff pastry production lines [41]. *S*. *warneri* species can be multidrug resistant, and this non-aureus *Staphylococcus* pathogen can cause various infections opportunistically [42].

Rustic potato bread 2 also contained *B. siamensis* and *B. subtilis*. Rustic potato breads 3 and 4 contained *B. amyloliquefaciens*, while rustic potato bread 4 also contained *B. methylotrophicus* and *B. firmus*. *Marinilactobacillus* spp. and *Micrococcus luteus* were detected in rustic potato bread 4. *B. amyloliquefaciens*, *B. methylotrophicus*, and *B. aryabhattai* were identified in sliced brown potato bread. In addition to *Bacillus* strains, *Streptococcus mitis* was also present. Rustic potato bread is a type of bread made with sourdough containing lactic acid bacteria. The carbohydrate, mainly starch and glucose fermenting *Marinilactobacillus* spp., has been detected in various fermented foods such as Cyprus sausages and table olives [43,44,45].

The crust of bran bread had a high viable cell count, and the bacteria identified from this type of bread was *B. methylotrophicus*. The viable cell count of sliced white bread was very high, and the bacteria identified were *Staphylococcus* spp., *S. warneri*, and *S. pasteuri*. Caro et al., 2023 [29], found similar strains of bacteria in different types of bread as *Micrococcus luteus* and *Staphylococcus*. The presence of *Staphylococcus* spp. in different types of bread was associated with inadequate human hygiene management and bread-making conditions.

The crust of bran bread had a high viable cell count, and the bacteria identified from this type of bread was *B. methylotrophicus*. The viable cell count of sliced white bread was very high, and the bacteria identified were *Staphylococcus* spp., *S. warneri*, and *S. pasteuri*. Caro et al., 2023 [29] found similar strains of bacteria in different types of bread as *Micrococcus luteus* and *Staphylococcus*. The presence of *Staphylococcus* spp. in different types of bread was associated with inadequate human hygiene management and bread-making conditions.

The source of *Bacillus* endospores probably is the soil and crops, which subsequently contaminate the flour [13]. Physicochemical properties of bread, such as temperatures above 25 °C combined with an a_w_ ≥ 0.95 and pH > 5, allow the growth of spore-forming bacteria. Ropy bread spoilage is caused by *Bacillus* strains due to proteolytic and amylolytic enzymes they produce. The endospores of these bacteria are heat stable and can survive baking in the center of the bread crumb. In addition to these, they are also resistant to desiccation, radiation, and different chemicals used in bread making. The prevalence of different spore-forming bacteria has been associated with the complex phenomenon of rope-forming *B*. *amyloliquefaciens*, *B. licheniformis*, and *B. subtilis* [7,16]. Our findings on the occurrence of some bacterial strains are consistent with previous studies. According to Vermelho et al., 2024 [13], the hygiene practices of the operators, the cleanliness of the production facilities, the origin and quality of the raw materials, and various other factors in the production process can potentially act as sources of microorganisms. Physical and chemical characteristics of bread support the growth of endospore-forming bacteria and molds [29]. In addition to adequate hygiene management, environmental factors such as humidity, oxygen, and temperature exposure can promote or suppress the growth of bacteria that may alter the quality of bread or pose a risk to consumer health.

## 4. Conclusions

The present study found that the microbiological quality of 30 breads varied. The total viable counts of bread crusts and bread crumbs differ significantly between breads. Bread crumb samples have lower cell counts in several cases compared to crust samples, but in samples with high total viable counts, crust and crumb showed similar results as in the case of sliced white bread. The K-means clustering algorithm results in four clusters. Bacterial growth and proliferation are also influenced by bread ingredients and not only by the processing environment and human factors. Despite the fact that hygienic indicator bacteria were absent and the total viable count did not exceed 400 CFU/g in highly contaminated breads (rustic potato bread and sliced white bread), *Aspergillus* spp., *Penicillium* spp., *S. aureus*, *S. pasteuri*, and *Streptococcus mitis* were present in different types of bread. The overall results indicate a need for improved hygiene and quality control measures in the bread production process for the benefit of public health, regulatory compliance, and consumer satisfaction.

## Figures and Tables

**Table 1:** Total viable counts of different bread crumb and crust samples (CFU/g).

	Region/Origin	Bread Crumb	Bread Crust
Bread Type		CFU/g	
Multigrain bread 1 pp	Harghita county	122.33 ± 39.55 ^abc^	164.00 ± 100.59 ^ab^
Multigrain bread 2 pp	Harghita county	10.00 ± 0.00 ^a^	44.67 ± 39.37 ^a^
Multigrain bread 3 wp	Harghita county	21.00 ± 13.45 ^a^	25.00 ± 13.08 ^a^
Multigrain bread 4 wp	Harghita county	12.00 ± 3.46 ^a^	31.33 ± 26.86 ^a^
Multigrain bread 5 wp	Harghita county	26.33 ± 7.51 ^a^	12.00 ± 3.46 ^a^
Multigrain bread 6 wp	Harghita county	113.00 ± 95.66 ^abc^	10.33 ± 0.58 ^a^
Rustic potato bread 1 pp	Harghita county	35.33 ± 27.15 ^a^	97.00 ± 50.59 ^a^
Rustic potato bread 2 wp	Covasna county	300.33 ± 178.01 ^d^	395.00 ± 52.43 ^b^
Rustic potato bread 3 pp	Harghita county	53.67 ± 16.86 ^a^	18.67 ± 15.01 ^a^
Rustic potato bread 4 pp	Harghita county	30.67 ± 10.60 ^a^	10.00 ± 0.00 ^a^
Rustic potato bread 5 wp	Harghita county	106.33 ± 110.59 ^abc^	15.67 ± 9.81 ^a^
Rustic potato bread 6 wp	Covasna county	11.67 ± 2.89 ^a^	58.67 ± 61.78 ^a^
Rustic potato bread 7 wp	Harghita county	38.00 ± 28.5 ^a^	17.67 ± 7.51 ^a^
Rustic potato bread 8 wp	Harghita county	18.00 ± 12.17 ^a^	16.00 ± 5.29 ^a^
Sliced white bread 1 pp	Harghita county	310.67 ± 94 ^d^	237.67 ± 45.36 ^ab^
White bread 2 wp	Harghita county	20.33 ± 11.93 ^a^	17.33 ± 12.70 ^a^
White bread 3 wp	Covasna county	14.33 ± 5.13 ^a^	10.67 ± 1.15 ^a^
Rye bread 1 pp	Harghita county	216.00 ± 41.94 ^cd^	239.00 ± 235.20 ^ab^
Rye bread 2 pp	Covasna county	54.67 ± 44.50 ^a^	10.00 ± 0.00 ^a^
Rye bread 3 pp	Harghita county	10.00 ± 0.00 ^a^	160.33 ± 122.21 ^ab^
Rye bread 4 pp	Harghita county	29.00 ± 24.02 ^a^	11.67 ± 2.89 ^a^
Sliced brown barley bread pp	Harghita county	22.67 ± 12.06 ^a^	207.67 ± 322.46 ^ab^
Sliced brown potato bread pp	Harghita county	66.00 ± 50.39 ^ab^	97.33 ± 126.18 ^a^
Peasant bread pp	Harghita county	10.00 ± 0.00 ^a^	12.67 ± 4.62 ^a^
Wheat germ bread pp	Harghita county	18.00 ± 3.00 ^a^	31.67 ± 37.53 ^a^
Wholemeal bread pp	Harghita county	14.00 ± 6.93 ^a^	17.67 ± 10.02 ^a^
Graham bread 1 wp	Harghita county	112.33 ± 54.50 ^abc^	145.00 ± 199.83 ^ab^
Graham bread 2 pp	Harghita county	206.00 ± 32.4 ^bcd^	22.67 ± 14.19 ^a^
Bran bread pp	Harghita county	10.00 ± 0.00 ^a^	134.00 ± 187.74 ^a^
French bread pp	Harghita county	20.00 ± 10.00 ^a^	10.33 ± 0.58 ^a^

^a,b,c,d^ Means ± standard deviation with different letters within rows, for each are significantly different by the Duncan’s test (*p* < 0.05). pp: pre-packed bread in bakery after baking; wp: unpacked bread, only packed in supermarkets.

**Table 2:** ANOVA table for K-means clustering analysis.

	Cluster		Error		F	Sig.
	Mean Square	df	Mean Square	df		
Bacteria count in bread crumbs	60,176.169	3	1080.839	26	55.675	0.000
Bacteria count in bread crusts	77,454.033	3	1101.137	26	70.340	0.000

**Table 3:** The ANOVA table for the post hoc test included the cluster number of cases.

	Sum of Squares	df	Mean Square	F	Sig.
Bacteria Count in Bread Crumbs Between Groups Within Groups Total	180,528.506	3	60,176.169	55.675	0.000
	28,101.802	26	1080.839		
	208,630.309	29			
Bacteria Count in Bread Crusts Between Groups Within Groups Total	232,362.099	3	77,454.033	70.340	0.000
	28,629.575	26	1101.137		
	260,991.674	29			

**Table 4:** Results of the identification of the bacterial strains isolated from the different bread samples.

Source of Isolation	Isolation Medium	Identified Closely Related Species Based on 16S rDNA	Sequence Similarity %
Multigrain bread 1	Nutrient Agar	*Chryseobacterium* spp.	99.00
Multigrain bread 1	Mannit–Kochsalz Agar	*Bacillus tequilensis*	99.68
Multigrain bread 1	Mannit–Kochsalz Agar	*Staphylococcus saprophyticus*	99.00
Multigrain bread 1	Czapek Dox Agar	*Bacillus subtilis*	99.00
Multigrain bread 1	Czapek Dox Agar	*Bacillus subtilis* subsp. *inaquosorum*	99.22
Bran bread	Cereus Selective Agar	*Bacillus methylotrophicus*	99.90
Rustic potato bread 1	Mannit–Kochsalz Agar	*Staphylococcus warneri*	99.90
Rustic potato bread 1	Mannit–Kochsalz Agar	*Bacillus siamensis*	99.40
Rustic potato bread 1	Czapek Dox Agar	*Bacillus amyloliquefaciens*	99.00
Rustic potato bread 1	Czapek Dox Agar	*Bacillus siamensis*	99.80
Rustic potato bread 2	Cereus Selective Agar	*Bacillus siamensis*	99.00
Rustic potato bread 2	Cereus Selective Agar	*Bacillus subtilis*	99.00
Rustic potato bread 3	Cereus Selective Agar	*Bacillus amyloliquefaciens*	99.00
Rustic potato bread 4	Mannit–Kochsalz Agar	*Marinilactobacillus* spp.	99.00
Rustic potato bread 4	Mannit–Kochsalz Agar	*Bacillus firmus*	99.00
Rustic potato bread 4	Cereus Selective Agar	*Micrococcus luteus*	100.00
Rustic potato bread 4	Cereus Selective Agar	*Bacillus methylotrophicus*	100.00
Rustic potato bread 4	Cereus Selective Agar	*Bacillus amyloliquefaciens*	99.00
Sliced brown potato bread	Mannit–Kochsalz Agar	*Streptococcus mitis*	90.00
Sliced brown potato bread	Mannit–Kochsalz Agar	*Bacillus aryabhattai*	99.79
Sliced brown potato bread	Cereus Selective Agar	*Bacillus methylotrophicus*	99.00
Sliced brown potato bread	Cereus Selective Agar	*Bacillus amyloliquefaciens*	99.00
Sliced brown potato bread	Cereus Selective Agar	*Bacillus amyloliquefaciens*	100.00
Sliced white bread	Mannit–Kochsalz Agar	*Staphylococcus warneri*	99.90
Sliced white bread	Mannit–Kochsalz Agar	*Staphylococcus pasteuri*	99.00
Sliced white bread	Mannit–Kochsalz Agar	*Staphylococcus* spp.	99.00
Rye bread 1	Mannit–Kochsalz Agar	*Staphylococcus saprophyticus*	99.00
Rye bread 1	Cereus Selective Agar	*Promicromonospora* spp.	99.00

## Data Availability

The original contributions presented in the study are included in the article, further inquiries can be directed to the corresponding author.
